# The His-tag as a decoy modulating preferred orientation in cryoEM

**DOI:** 10.3389/fmolb.2022.912072

**Published:** 2022-10-17

**Authors:** Raquel Bromberg, Kai Cai, Yirui Guo, Daniel Plymire, Tabitha Emde, Maciej Puzio, Dominika Borek, Zbyszek Otwinowski

**Affiliations:** ^1^ Department of Biophysics, The University of Texas Southwestern Medical Center, Dallas, TX, United States; ^2^ Ligo Analytics, Dallas, TX, United States; ^3^ Department of Biochemistry, The University of Texas Southwestern Medical Center, Dallas, TX, United States; ^4^ Center for Structural Genomics of Infectious Diseases, Dallas, TX, United States

**Keywords:** cryo-electron microscopy single particle reconstruction, cryoEM SPR, air-water interface, AWI, His-tag, preferred orientation, visualization

## Abstract

The His-tag is a widely used affinity tag that facilitates purification by means of affinity chromatography of recombinant proteins for functional and structural studies. We show here that His-tag presence affects how coproheme decarboxylase interacts with the air-water interface during grid preparation for cryoEM. Depending on His-tag presence or absence, we observe significant changes in patterns of preferred orientation. Our analysis of particle orientations suggests that His-tag presence can mask the hydrophobic and hydrophilic patches on a protein’s surface that mediate the interactions with the air-water interface, while the hydrophobic linker between a His-tag and the coding sequence of the protein may enhance other interactions with the air-water interface. Our observations suggest that tagging, including rational design of the linkers between an affinity tag and a protein of interest, offer a promising approach to modulating interactions with the air-water interface.

## 1 Introduction

Cryogenic electron microscopy single particle reconstruction (cryoEM SPR) datasets consist of images of macromolecular particles dispersed in a thin layer of amorphous ice formed over a thin supporting material that is attached to a metal grid. In recent years, there have been several studies on the angular and positional orientations of particles within the ice layer in cryoEM SPR and on the interactions of particles with the air-water interface (AWI) during cryo-cooling. In these studies, the kinetic parameters, variations in mechanical support, and chemical modifications to the support were explored. A number of strategies helping to keep biological samples in their optimal state have been proposed (D'Imprima et al., 2019; Glaeser, 2018; Noble et al., 2018a; Drulyte et al., 2018).

The AWI has been shown (D'Imprima et al., 2019; Noble et al., 2018b; Chen et al., 2019) to have a damaging influence due to the extremely high hydrophobicity of air (van Oss et al., 2005). Many macromolecular particles have on their surface hydrophobic patches which are strongly attracted to the AWI (Taylor and Glaeser, 2008; Glaeser and Han, 2017), and diffusion allows particles to reach the interface within milliseconds, with partial or full unfolding frequently following initial binding (D'Imprima et al., 2019; Noble et al., 2018b; Glaeser and Han, 2017). Even if molecules do not unfold, they may become preferentially oriented (Han et al., 2012; Tan et al., 2017; Noble et al., 2018b; Chen et al., 2019). The binding to the AWI has also a significant advantage in reducing particle exclusion from thin ice, which is highly preferred for obtaining high quality data. Therefore, interactions between particles and the AWI have a complex impact upon experimental results.

Preferred orientation hinders cryoEM SPR reconstruction in two ways (Radermacher, 1988). Firstly, the preferred orientation results in a systematic lack of information regarding some orientations through the so-called missing cone effect, which leads to uneven coverage of reciprocal space and thus can be considered analogous to anisotropic diffraction in X-ray crystallography. In addition, the lack of a large group of orientations affects the convergence of the computational procedures used in 3D reconstruction for cryoEM SPR. Particularly for macromolecular systems having molecular mass lower than ∼150 kDa, systematic misalignment of particles will result in introducing bias and artifacts to the reconstruction (Radermacher, 1988; Barth et al., 1989; Naydenova and Russo, 2017; Tan et al., 2017; Noble et al., 2018b).

Several approaches have been proposed to alleviate the problem of damage induced by the AWI or of the preferred orientation induced by it. They include using surfactants (Frederik et al., 1989; Glaeser et al., 2016; Chen et al., 2019) to saturate the surface at the AWI or the surface of the support (Russo and Passmore, 2014), and graphene oxide or graphene supports to prevent interactions of particles with the AWI (Pantelic et al., 2010; Pantelic et al., 2011; Wang et al., 2020a; Wang et al., 2020b). These supports may also be chemically modified to promote specific, high-affinity interactions with particles (Crucifix et al., 2004; Kelly et al., 2008; Kelly et al., 2010a; Kelly et al., 2010b; Han et al., 2012; Llaguno et al., 2014; Benjamin et al., 2016a; Benjamin et al., 2016b; Wang et al., 2020a; Wang et al., 2020b; Yeates et al., 2020). Finally, fast new approaches for depositing samples on a grid have been developed (Arnold et al., 2017; Dandey et al., 2018; Schmidli et al., 2018; Wei et al., 2018; Ravelli et al., 2019; Dandey et al., 2020). One of the strategies for changing interactions of macromolecules with a support, and with the AWI, is to chemically modify the molecule itself without changing the chemical properties of the support. Affinity tags chemically modify a protein and have been used in sample preparation for cryoEM in the context of affinity purification (Benjamin et al., 2016a; Benjamin et al., 2016b; Wang et al., 2020a; Wang et al., 2020b), but as we observed, tag presence can modify interactions with the AWI even without the use of affinity grids.

The His-tag is an affinity tag used widely to facilitate purification of recombinant proteins to homogeneity (Hochuli et al., 1988). Such proteins, with 6–10 consecutive histidine residues inside the tag which is introduced at their N- or C-termini, are overexpressed, with the tag facilitating purification by immobilized metal ion affinity chromatography (IMAC) (Wong et al., 1991). After purification, the His-tag is usually cleaved with a protease that recognizes the specific amino acid sequence motif—a cleavage site. Several proteolytic enzymes are used for this purpose, e.g. thrombin, factor Xa, Tobacco Etch Virus (TEV)-protease, and carboxypeptidase A. TEV-protease is a common choice because its classical cleavage site ENLYFQ(G/S) is very specific; the cleavage is performed after a glutamine residue, with TEV cleavage generating only a single amino acid addition if the His-tag was used at the N-terminal end of the expressed protein. When used for this purpose, TEV protease is usually expressed with an uncleavable His-tag (Raran-Kurussi et al., 2017). After the first round of purification, when a His-tagged protein of interest is separated from untagged proteins using IMAC, His-tag labelled TEV protease is added to the protein of interest. TEV protease cleaves His-tags from the protein of interest, and this cleavage is followed by a second round of IMAC to capture both the His-tag labelled TEV protease and also molecules with uncleaved His-tags, while the His-tag free protein is collected in the flow-through fractions. This procedure is frequently followed by an additional chromatography step, e.g. size exclusion chromatography, to assure that the protein is properly folded and in its proper oligomeric state.

The presence of the His-tag in crystallization is considered an obstacle, as the His-tag is usually connected to the protein of interest by a flexible linker and the increased flexibility may interfere with crystallization success (Majorek et al., 2014). However, in the case of cryoEM SPR, this does not necessarily pose a problem as the requirements for conformational homogeneity for a cryoEM sample are less stringent than for crystallization. With multiple possible conformations of flexible sequence extensions, forming a crystal lattice may be a challenge, and a well-packed crystal lattice is necessary for high-resolution diffraction. In cryoEM SPR, flexible sequence extensions will not contribute to the alignment of particles if they are truly flexible and will produce the 3D reconstructions with the flexible parts averaged out. However, such flexible extension may sometimes contribute to aggregation in the case of a cryoEM sample or may have higher propensity to unfolding at the AWI in comparison with more compact structures. Here we show and discuss how the His-tag affects interactions between labelled proteins and the AWI, which results in the modulation of particle preferred orientations.

## 2 Materials and methods

### 2.1 Protein expression and purification

Coproheme decarboxylase from *Geobacillus stearothermophilus* (HemQ) is encoded by the GYMC52_3505 plasmid which is available from the DNASU Plasmid Repository (https://dnasu.org/). The open reading frame of the protein is cloned in the pMCSG7 vector containing Tobacco Etch Virus (TEV) cleavable N-terminal His6-tag (Stols et al., 2002).

His-tagged Tobacco Etch Virus (TEV) protease encoded by the pMHTDelta238 plasmid is available from the DNASU Plasmid Repository (https://dnasu.org/). It expresses a mutated and truncated form of the TEV protease as an N-terminal fusion to MBP-His7 (Raran-Kurussi et al., 2017), in which the MBP fusion is removed *in vivo* by autocleavage, leaving His7-TEV.

Our protein expression and purification followed a previously established protocol (Kim et al., 2011) with modifications that we describe here in detail.

The GYMC52_3505 and pMHTDelta238 plasmids were transformed to Rosetta2 (DE3)pLysS competent cells (Cat. No. 71401–3, EMD Millipore). Transformation reactions were spread on LB plates with ampicillin (Amp) at 200 µg/ml and chloramphenicol (Cam) at 37 µg/ml for GYMC52_3505, and with kanamycin (Kan) at 25 µg/ml and Cam at 37 µg/ml for pMHTDelta238. Single colonies grown on selective LB plates were used to initiate 3 ml or 25 ml liquid media cultures of Luria Broth medium (LB) with 200 µg/ml Amp and 37 µg/ml Cam for coproheme decarboxylase, and with 50 µg/ml Kan and 37 µg/ml Cam for TEV. All cultures were grown overnight in an incubated shaker (225 rpm, 37°C). The next morning, these cultures were used to seed larger volume cultures at the ratio 1:1000. 1 L or 6 L LB cultures, with appropriate antibiotics, were grown at 37°C with 225 rpm shaking until OD_600_ of ∼1.0, when the expression was induced by adding Isopropyl β-d-1-thiogalactopyranoside (IPTG) to the final concentration of 1 mM. The temperature in the shaker was decreased to 28°C for coproheme decarboxylase and 20°C for TEV, and cultures were grown overnight at these temperatures with shaking at 225 rpm. The cultures were centrifuged, and pellets were further processed and purified with slightly different protocols.

The bacterial pellets were resuspended in the lysis buffer (50 mM sodium phosphate pH 7.5, 300 mM NaCl buffer) at ∼5 ml of the buffer per 1 g of the bacterial pellet. One tablet of cOmplete™ Protease Inhibitor Cocktail (Roche) was added to lysate. The bacterial suspension was then sonicated on ice for 15 min [30 × (30/30 s on/off), at 50% amplitude]. The lysate was centrifuged at 45,000×g for 30 min. The supernatant was retained and clarified by filtration through the 0.8 µm filter Millex-AA Syringe Filter Uni (MCE/blue colour). The clarified lysate was applied onto the column containing 3–4 ml of Talon resin preequilibrated with a binding buffer consisting of 50 mM sodium phosphate pH 7.5 and 300 mM NaCl. After the lysate flowed gravitationally through the column, ∼100 ml of the binding buffer was applied to remove non-specifically bound proteins. The protein was eluted from the column in 1.5–2 ml fractions by applying ∼15 ml of the elution buffer consisting of 50 mM sodium phosphate pH 7.5, 300 mM NaCl, and 150 mM imidazole. The aliquot of lysate as well as aliquots of the flow-through fraction, a fraction from the wash with binding buffer and a fraction from the wash with wash buffer, and each of the elution fractions were analyzed with the SDS-PAGE (Supplementary Figure S1). The fractions containing coproheme decarboxylase or TEV proteins were pooled. Purified according to the above protocol, His-tagged TEV protease (∼1 mg) was added to pooled fractions of coproheme decarboxylase proteins and dialyzed 24–48 h against the binding buffer for HemQ preparations with completely or partially cleaved His-tag. Then, for samples with completely cleaved His-tag, dialysate was applied to 5 ml of Talon resin equilibrated with the binding buffer and flow-through was collected. The samples with partially cleaved His-tag were not subjected to the second Talon column. The dialysate for samples with the His-tag partially cleaved, or flow-through from either first or second round of affinity purification for samples with all His-tags cleaved or all His-tags present were concentrated to ∼2 ml with Amicon® Ultra-15 Centrifugal Filter Unit with Ultracel® regenerated cellulose membrane and a 10 kDa molecular mass cutoff (Millipore), filtered through a 0.2 µm centrifugal filter and applied to a Superdex® 200 10/300 column run with an AKTA Pure system at a flow rate of 1 ml/min in 50 mM HEPES pH 7.5, 100 mM NaCl. Coproheme decarboxylase eluted at ∼12 ml, in agreement with the molecular mass of a pentamer (∼144–175 kDa depending on His-tag status) (Supplementary Figure S2). The fractions were collected, analyzed on the SDS-PAGE and concentrated with ∼2 ml with Amicon® Ultra-15 Centrifugal Filter Unit with Ultracel® regenerated cellulose membrane and a 10 kDa molecular mass cutoff (Millipore) to the desired concentrations (20–30 mg/ml), which were assessed by measuring UV absorbance at 280 nm. In addition, centrifugal filtration was accompanied with buffer exchange to 50 mM HEPES pH 7.5, 100 mM NaCl. For partially cleaved and uncleaved batches of the protein, we did not use the second IMAC step, and instead applied the sample after TEV digestion directly to the gel filtration column, with the assumption that it contained a mixture of tagged states in the pentamer for a partially cleaved protein.

### 2.2 Preparation of grids, cryoEM data collection and data analysis

The purified coproheme decarboxylase samples were used to prepare grids. We used gold Quantifoil R 1.2/1.3 grids. The grids were glow discharged 90 s at 30 mA with a PELCO easiGlow™ Glow Discharge Cleaning System to obtain a hydrophilic surface. The glow-discharged grids were used to prepare vitrified samples with the Thermo Scientific Vitrobot Mark IV System. We applied 3 µl of purified protein to the glow-discharged surface of the grid at 4°C, 100% of humidity and blotted the solution for 5.5–6 s with blot force of either 19 or 20.

The data were acquired and analyzed as described before (Bromberg et al., 2020) (Table 1). Briefly, the cryoEM datasets for HemQ without His-tag (HemQ-57K), with His-tag partially present (HemQ-45K-T) were collected with a 200 kV Talos Arctica using a K2 Gatan camera run in super-resolution mode, with a physical pixel of 0.72 Å. A phase plate was not used and the objective aperture was not inserted. For HemQ-57K, 268 movies were collected with an exposure time of 40 s/movie. Each movie contains 100 frames with an exposure time of 0.4 s/frame and an electron dose of 90 e/Å^2^ per movie. For HemQ-45K-T, 28 movies were collected with an exposure of 80 s/movie. Each movie contained 200 frames with an exposure time of 0.4 s/frame and an electron dose of 180 e/Å^2^ per movie. The dataset for HemQ with His-tag present in all copies (HemQ-45K-K) was acquired with a 300 kV Titan Krios and K3 Gatan camera run in super-resolution mode, with a physical pixel of 0.835 Å. A phase plate was not used and the objective aperture was not inserted, the energy filter was used with slits set at 25 eV. For HemQ-45K-K, 937 movies were collected with an exposure of 7 s/movie. Each movie contained 100 frames with an exposure time of 0.07 s/frame and an electron dose of 78 e/Å^2^ per movie.

**TABLE 1:** Data collection and processing.

	HemQ-57K (no His-tag)	HemQ-45K-T (partial His-tag)	HemQ-45K-K (His-tag present)
Instrument	Talos 200 kV	Talos 200 kV	Krios 300 kV
Detector	K2	K2	K3
Energy filter	No	No	Yes
Objective aperture	No	No	No
Nominal magnification	57,000×	45,000×	105,000×
Data collection mode	Parallel beam; 1 hole per movie	Parallel beam; 1 hole per movie	Beam-image shift; 3×3 holes with beam tilt/astigmatism compensation
Frames per movie	100	200	100
Electron dose (e/A^2^/frame)	0.9	0.9	0.78
Exposure time (s/frame)	0.4	0.4	0.07
Super-resolution mode	Yes	Yes	Yes
Detector pixel size (Å)	0.72	0.91	0.835
Data pixel size (Å)	0.36	0.455	0.417
Movies acquired	268	28	937
Movies used for processing	258	28	937
Molecular weight (kDa)	145	145	157
Reconstruction symmetry	C5/C1	C5/C1	C5/C1
Total picked particles	156,210	38,818	1,098,210
Particles after 2D averaging	145,966	38,818	1,041,821
Particles used in refinement	81,302	38,818	1,041,821
Resolution FSC_0.143_ [C5/C1, Å]	2.32/2.93	2.53/∼4.00	2.46/3.68

We processed all datasets with cisTEM (Grant et al., 2018) with C5 symmetry applied. We modified the cisTEM pipeline by adding reference-based refinement of aberrations, including coma and trefoil, as in JSPR and Relion (Zivanov et al., 2018; Li et al., 2019). All the structures were solved with MOLREP (Vagin and Teplyakov, 2010) used within CCPEM (Wood et al., 2015; Burnley et al., 2017; Nicholls et al., 2018) with 6VSA.pdb used as a search model. Coot (Emsley and Cowtan, 2004; Emsley et al., 2010) was used to manually inspect the models and maps.

For reconstruction with C1 symmetry of the dataset with all His-tags included, we used cryoSPARC (Punjani et al., 2017). The particle stack (1,041,821 particles, box size 256) was exported from cisTEM into Relion format. The exported particle stack was imported to cryoSPARC using Import Particles job. An *ab initio* reconstruction job was then conducted in C1 symmetry with three *ab initio* classes to minimize biases. One out of three *ab initio* classes (consisting of 616,955 particles) showed appropriate representation of all views of the protein and was selected for the next processing step. One round of homogeneous refinement in C1 symmetry using the selected *ab initio* model and all particles (1,041,821 particles) resulted in a map with 3.68 Å resolution.

For tomographic reconstruction, IMOD software (Kremer et al., 1996) was used to align the tilt serial images and to reconstruct the tomograms by the weighted back-projection (WBP) approach. Ice thickness measurements were performed as previously described (Noble et al., 2018b). Briefly, after orienting the tomograms in IMOD to make the AWI parallel to the field of view, two adsorbed particle layers were identified and used to locate the two AWIs, and the distance between the two interfaces was measured as the ice thickness.

Figures were prepared with PYMOL (The PyMOL Molecular Graphics System, Version 2.3.2, Schrödinger, LLC.), Excel (Microsoft Office 365 ProPlus, Microsoft), and Adobe Illustrator (Adobe Illustrator 2020; Version 24.0.1, Adobe Inc.). PYMOL includes an APBS electrostatic plugin (Baker et al., 2001), which we used with default values an input generated with 6VSA.pdb. Electrostatic surfaces are displayed at ± 10 k_B_T/e^−^ at full color saturation.

## 3 Results and discussion

**FIGURE 1:**
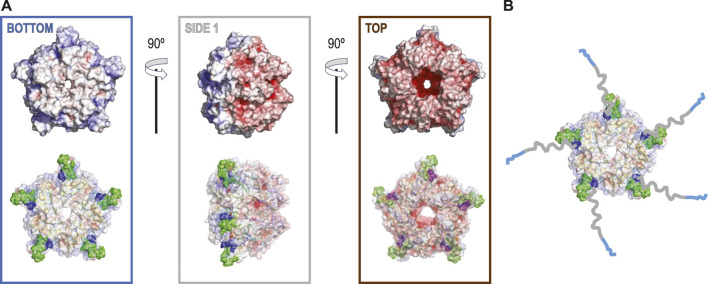
**(A)** The electrostatic potential map for 6VSA.pdb, which represents the cryoEM reconstruction of the cleaved version of coproheme decarboxylase. Three different orientations that stress a highly polarized charge distribution for this protein that explains observed patterns of preferred orientation (top). In the bottom row, each orientation of the electrostatic potential map is shown with 40% of transparency to show the orientations of the hydrophobic loop between residues 110–120 (green spheres) based on 1T0T.pdb and its proximity to the N-terminus (dark blue speres). **(B)** The model with the His-tags and their linkers added to show how they can extend from the surface of the protein up to ∼60–65 Å.

**FIGURE 2:**
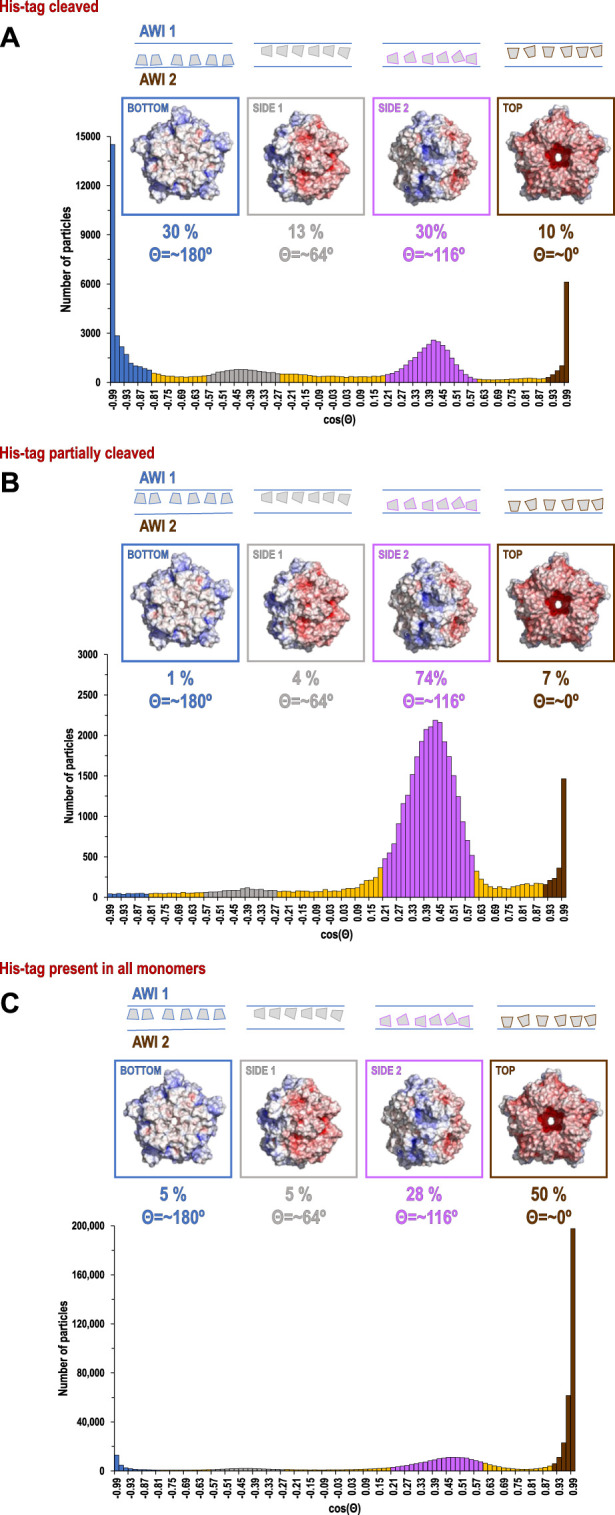
Histograms of distributions for particles of coproheme decarboxylase with His-tag cleaved **(A)**, His-tag partially cleaved **(B)**, and His-tag uncleaved **(C)**. The histograms show the number of particles as the function of cosine Θ, where Θ is the angle between the 5-fold axis of the particle and the direction of the beam. The map of the electrostatic potential for each orientation is shown above the specific peaks corresponding to the most frequent orientations.

**FIGURE 3:**
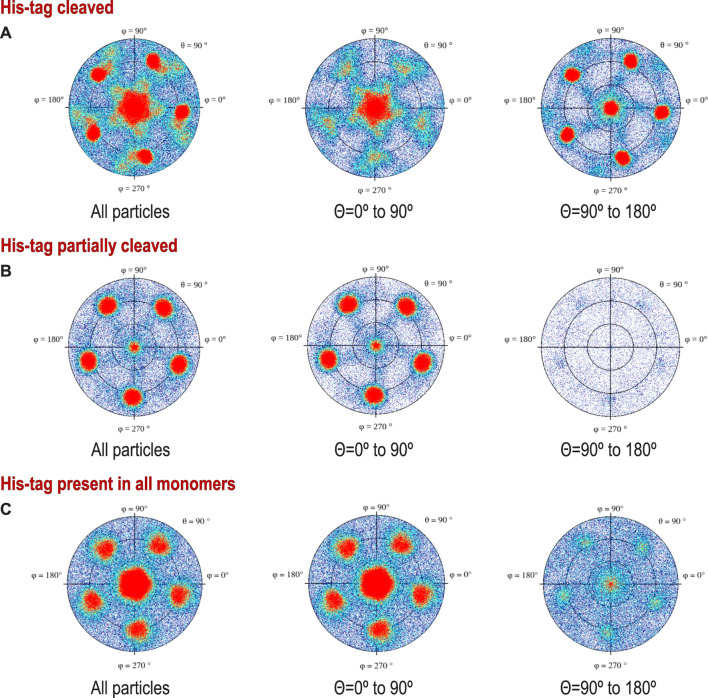
Preferred orientations (heat plots) shown in polar coordinates for protein with: **(A)** His-tag cleaved protein, **(B)** His-tag partially cleaved, and **(C)** His-tag uncleaved. To stress the difference in the polarity of the particles, we show on the left traditional plots for all particles, while the middle and right figures show (+) and (-) polarities (particles tilted away from or toward the beam). The different polarities result from different angles of interactions with the AWI, but also may result from interactions with two separate AWIs, which would symmetrize the histogram, as the cosine of the angle between the particle and the beam changes sign for the same geometric interaction with the AWI. We do not directly know how much these two effects contribute to our data.

Coproheme decarboxylase (formerly HemQ) from *Geobacillus stearothermophilus* was one of the targets of the Midwest Center for Structural Genomics (MCSG; target APC35880). We solved its X-ray crystallographic structure in 2004 (PDB code: 1T0T.pdb) (Stols et al., 2002; Kim et al., 2011), while others determined its function later. We recently characterized this protein using cryoEM SPR (EMPIAR-10363, EMD-21373 and EMPIAR-10362, EMD-21376) (Grant et al., 2018; Bromberg et al., 2020) and noticed during cryoEM experiments that batches of the protein purified at different times showed different patterns of preferred orientation during cryoEM SPR data collection (Figure 1 ‐3, Supplementary Figure S3). This observation prompted the analysis presented here. In the process of analyzing these datasets, we acquired additional cryoEM SPR datasets (Table 1).

Our data processing and analysis indicate that His-tag presence and occupancy greatly change preferred orientation patterns (Figures 2A–C) of 5-fold symmetric coproheme decarboxylase particles. Without the His-tag, these particles show very strong preferred orientation, with most (∼40%) having their 5-fold axis oriented along the electron beam and perpendicular to both AWIs. However, out of two possible polarities (Figure 2A and Figure 3A), one is preferred (30% *vs*. 10%). With the His-tag partially cleaved, more particles are rotated on their side, which significantly changes the patterns of preferred orientation; ∼78% of the particles have their 5-fold axes oriented at ∼116^°^ or equivalently, at ∼64^°^ (if the opposite orientation of the symmetry axis is used) to the AWI (Figures 2B, 3B). Finally, when the His-tag is present in all monomers of the particle, particles have also very strong preferred orientation with ∼55% of particles having their 5-fold axis oriented roughly parallel to the beam again but with the reversed polarity in comparison with the dataset where all particles have His-tag cleaved (Figures 2C, 3C).

We present these data using a new graphical representation for preferred orientation of particles with n-fold symmetry (Figure 2), in addition to standard angular plots (Figure 3). 2D orientation plots of different types are not straightforward to interpret quantitatively. For this reason, we projected them on a single axis, which is the angle between the electron beam and symmetry axis (Θ). Because particles of coproheme decarboxylase are polar, we can differentiate between parallel and anti-parallel orientations. The choice regarding which orientations are considered parallel is arbitrary, but we kept it consistent in our figures to facilitate comparisons. For such 1D orientation plots, uniform angular sampling would result in a sine modulation. We compensated for it by presenting the plots in uniform steps of cos(Θ). A random angular distribution will be flat in such a representation. The extremes in the range of cos(Θ) represent parallel and antiparallel to the beam orientation of particles. This representation is natural for particles with n-fold symmetry, but there is no natural choice of Θ for particles without symmetry.

The data show only the orientation with respect to the beam, including the polarity aspect. However, if we are interested in polarity of orientation with respect to the AWI, we need to consider the possibility of particles that bound to both AWIs in a thin layer to be cryo-cooled. The strong polarity pattern, i.e. the observed asymmetry (Figures 3A,C), indicates that the frequency of binding to these two interfaces is very different. Precisely establishing the apolar binding pattern for a single AWI would require tomographic reconstruction to isolate data arising from each interface. We performed such reconstruction for one of our datasets for a sample with His-tag uncleaved (Figure 5) and the observed asymmetry of binding agrees with quantitative data presented on Figure 2C and Figure 3C for the same type of the sample. Past tomographic reconstruction of particles trapped in a thin ice layer established that typically there is strong asymmetry in binding to the top and bottom (relative to the beam direction) interfaces (Tan et al., 2014; Noble et al., 2018b; Klebl et al., 2020), and this is also fully consistent with our data.

For polar particles, an advantage of our method of presenting data is that it provides a quick and convenient characterization of asymmetry between two surfaces, and it also allows for retrospective analysis of cryoEM SPR data. The width of the peaks in our histogram (Figure 2) results not only from the range of the angles between particles and the AWI, but also from variations in the tilt between the AWI and the beam. For typical grids, this is on the order of a few degrees (Noble et al., 2018b) when data are acquired at a zero-tilt angle. In addition to traditional 2D frequency plots, we also calculated 2D plots for two opposing polarities, to emphasize the significance of the asymmetry of binding to the AWI in two opposing directions (Figure 3).

The direction of preferred orientation can strongly influence 3D reconstruction; if it is aligned with the symmetry axis, as it is for datasets with the His-tag cleaved in all copies or the His-tag present in all copies, it generates only one back projection orientation, while if it is at a high angle to the symmetry axis as it happens for His-tag partially cleaved, it generates a number of back projections equal to the symmetry factor (5 in this case), all contributing to the 3D reconstruction. Therefore, the change from one pattern of preferred orientation to another can have high impact, even if the orientations are not uniformly sampled.

The significant change in patterns of preferred orientation between a protein with and without a His-tag prompted us to analyze 6VSA.pdb (cryoEM data without His-tag) and 1T0T.pdb (X-ray data without His-tag) to identify possible reasons for the observed rearrangements.

The coproheme decarboxylase molecule is a compact structure which has a strikingly asymmetric charge distribution, with only a small number of possible hydrophobic patches that would be attracted by the AWI (Figure 1A). One of the patches is located at the bottom of the pentamer (Figure 1A) and this patch most likely facilitates preferred orientation for proteins without His-tags (Figure 2A). However, monomers retaining the His-tag had their N-termini extended by the sequence, HHHHHHSSGVDLGTENLYFQSNA, 23 amino acids in length, and originating from the MCSG7 vector. This part of the structure had no reconstructed density in our reconstructions performed with C5 symmetry, as expected for parts of the chain that are disordered. This disordered sequence can extend up to ∼3.25 Å per amino acid residue, so it can extend up to ∼60–65 Å, which is more than the radius of the reconstructed part of the protein structure. The N-terminal tail containing His-tag may dynamically bind the flat, hydrophobic patch at the bottom of the pentameric assembly, and so prevent binding of this hydrophobic patch to the AWI. It may bind directly to the AWI as its parts have hydrophobic character. The stretch of histidine residues in the His-tag can balance highly negative charges on the other side of the molecule, and form a new neutral/hydrophobic surface that may preferentially interact with the AWI. Finally, the N-terminal tail with the His-tag and its linker may interact with the hydrophobic loop discussed below and enhance its interactions with the AWI.

**FIGURE 4:**
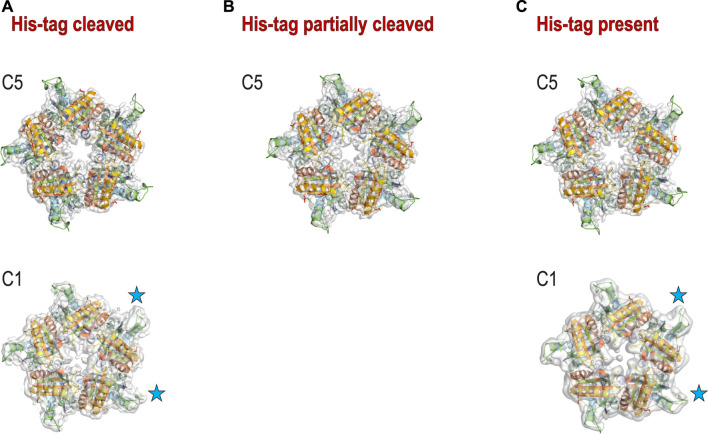
3D reconstructions for three samples. **(A)** 3D reconstruction with C5 (top) and C1 (bottom) symmetries for the sample with His-tag cleaved; **(B)** 3D reconstruction with C5 symmetry for the sample with His-tag partially cleaved. The reconstruction with C1 symmetry did not provide the interpretable density. **(C)** 3D reconstruction with C5 (top) and C1 (bottom) symmetries for the sample with His-tag uncleaved. The blue stars denote the 2 loops that have the best density in C1 reconstructions.

The second hydrophobic patch is located at a highly hydrophobic surface loop between amino acids 110 and 120 (PAYSYVSVVEL) (Figure 1). This region was ordered in the X-ray structure (1T0T.pdb) due to stabilizing interactions provided by the crystal lattice and a polyethylene glycol molecule present in crystallization solution, but in the cryoEM reconstructions obtained in C5 symmetry in this study (with and without His-tag), it is not visible. This unstructured region in our reconstruction is likely to serve as the AWI anchor and thus becomes partially unfolded (D'Imprima et al., 2019; Glaeser and Han, 2017), while the compactness of the rest of the structure prevents further unfolding. If the region was only unfolded upon binding to the AWI, then only 2 out of 5 loops in the pentamer would be affected, with the remaining 3 providing a weak but unambiguously visible contribution. This loop seems to direct the orientation with respect to the AWI both for fully His-tagged particles and those with the His-tag entirely cleaved with similar frequency (Figures 2A,C). The N-termini of each monomer are also close to this surface loop. The much stronger presence of the side orientation in datasets with partially cleaved His-tag (Figure 2B) may be due to the AWI interacting synergistically with both the hydrophobic part of the His-tag and the surface loop. We could not test these hypotheses for reconstructions performed with C5 symmetry and all datasets were reconstructed with C5 symmetry because we expected that the reconstruction in C1 would be severely affected by very strong preferred orientation. However, the observed differences in patterns of preferred orientation, in particular the reversal of polarity for the dataset with His-tag entirely uncleaved, prompted us to attempt reconstruction in C1. We succeeded in finding the self-consistent group of orientations for particles in all three datasets, i.e. particles aligned to each other in 2D. However, the 3D reconstruction produced interpretable maps only for the dataset without the His-tag and with the His-tag present in all subunits, with resulting resolution of reconstructions of 2.93 and 3.68 Å (by FSC_0.143_ criterion), respectively (Table 1). Although we successfully performed classification for the dataset with partially cleaved His-tag, we were unable to obtain 3D reconstruction, despite the FSC_0.143_ indicating a resolution of ∼4 Å. The resolution of the reconstructions in C1 is lower than for reconstructions in C5, as expected, but we can analyze structural differences. The His-tag is not visible in the reconstruction from the sample with all monomers containing His-tags. The partially ordered loops in the reconstructions for the sample with His-tags and the sample without His-tag assume conformations similar to the conformations observed in the X-ray crystal structure (1T0T.pdb) (Figure 4). We believe that reconstructions in C1 are partially symmetrized, i.e. have approximately C5 symmetry due to particles having strong preferred orientation, with properties facilitating such symmetrization. HemQ is a pentamer and all three datasets we analyzed show preferred orientation. In two datasets, the 5-fold axis of HemQ is roughly parallel to the beam direction for most of the particles, with groups of particles having opposite polarities (Figures 2A, 3A and Figures 2C, 3C). For the third dataset, for particles with His-tag partially cleaved, the 5-fold axis is oriented ∼116° with respect to the beam direction for most of the particles (Figures 2B, 3B). These patterns of preferred orientations have specific consequences depending on the symmetry used in reconstruction. The preferred orientation with the 5-fold symmetry axis parallel to the beam direction is detrimental because little new information is generated after C5 symmetry expansion is applied to particle orientations. For the preferred orientation with 5-fold symmetry at a ∼116° angle to the beam direction, the C5 symmetry expansion results in new orientations, so the reconstruction in C5 works well, even though the preferred orientation was much stronger for this dataset, and we also had a limited number of particles (Table 1).

The success of reconstruction in C1 for particles having their 5-fold axis oriented along the beam depends on the signal strength of the features differentiating monomers. The differences between the monomers must be significant enough so that the particles can align asymmetrically, otherwise the reconstruction in C1 will be averaged in an approximate C5 symmetry even though this symmetry has not been explicitly applied. We believe that although we successfully reconstructed two datasets in C1, the symmetrization took place in both cases due to the very low mass of features we tried to discern (1–2 kDa). Nevertheless, we observe that three out of five hydrophobic loops show density that is much better ordered than the density for the remaining two loops (Figure 4). This is consistent with the hypothesis that these two less ordered loops interact with the AWI for datasets both with and without His-tag.

**FIGURE 5:**
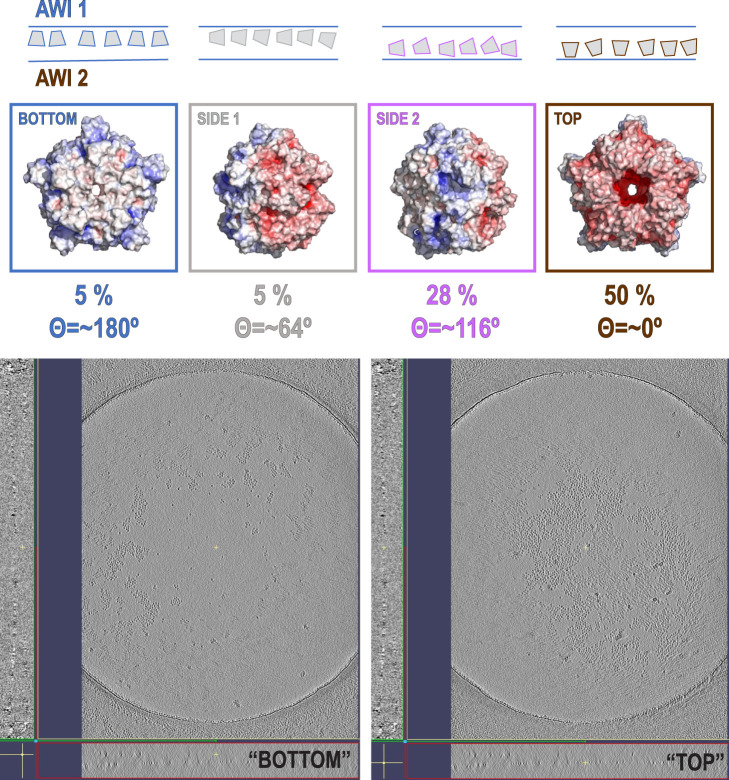
Tomographic reconstruction of the sample with His-tag present in all copies. The upper part of the figure shows the distribution of orientations for that sample, while the two panels at the bottom part of the figure represent the patterns on two different AWIs. One of the AWIs (left) shows very few particles in comparison with the second AWI (right). This is consistent with the results of our data analysis performed on a different subset of particles from the same type of sample. The particles in the sample are also forming “groups” that are not present in the sample with His-tags cleaved (Supplementary Figure S3A–C).

What would then cause the reversed polarity between the datasets with the His-tag in all monomers and with the His-tag entirely cleaved? Our initial hypothesis was that His-tag presence may obscure the hydrophobic surface on one of the sides of HemQ (Figure 1A). However, the datasets with the His-tag entirely uncleaved suggests a different model. The particles in this dataset (Figure 5 and Supplementary Figure S3) group together but do not aggregate and the reversed polarity of their preferred orientation (Figures 2C, 3C) indicates that the highly charged end of the molecule interacts with the AWI. A possible explanation for such interactions is that the positively charged end of the His-tag compensates the negative charges on the surface of the molecule. The grouping that we observe (Figure 5 and Supplementary Figure S3) is consistent with this hypothesis, as the His-tag with its linker in its extended conformation is long enough (∼5–6 nm) to capture and interact with the negatively charged surface of the same particle but can also interact with the surfaces of other particles. Such interactions would generate the “grouping” we observed in Figure 5.

**FIGURE 6:**
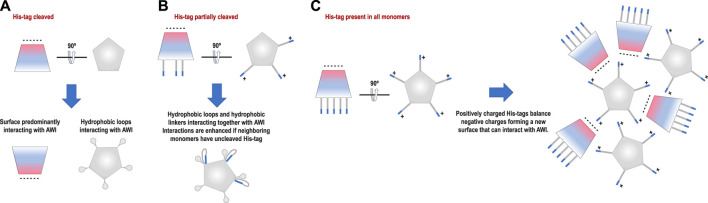
Proposed models of interactions with the AWI for each sample. **(A)** Particles with His-tag cleaved interact with the AWI using predominantly a hydrophobic “bottom” surface. **(B)** Particles with His-tag partially cleaved interact with the AWI using a hydrophobic loop with interactions most likely enhanced by the hydrophobic linker between the N-terminus of the protein and the His-tag. **(C)** Particles with His-tag present in all monomers interact with the AWI using the predominantly negatively charged side of the molecule. This can be achieved if the positively charged His-tag compensates the negative charge on the other surface, forming a new interface that can interact with the AWI.

The His-tag may act as a decoy compensating for the charge at one end of the particle to form a new surface interacting with the AWI or as a decoy obscuring the more hydrophobic, flat surface perpendicular to the 5-fold axis of the particle to prevent interactions with the AWI. The linker between the N-terminus of the protein and the His-tag may enhance the hydrophobicity of the already hydrophobic loop. These effects together are likely creating the observed distributions (Figures 5, 6). There is no doubt that the interactions with the AWI have been also affected by our attempts to obtain as thin an ice layer as possible. However, for particles having limited molecular mass, thin ice is required to achieve a high resolution reconstruction. Particles are suspended at different z values, i.e. they will have different defocus in thicker ice and so one of the steps in refinement needs to include per particle defocus refinement. Unfortunately, for particles that have lower molecular mass, the signal-to-noise ratio (SNR) is too low for this step to succeed and without this refinement reconstruction to high resolution is not possible. Thin ice results in more uniform defocus per particle and an SNR that is higher, so per-particle defocus refinement becomes feasible for a higher number of projects.

**FIGURE 7:**
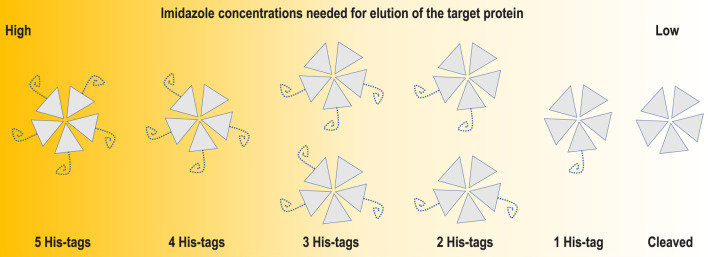
Schematic representation of an incomplete cleavage problem for a pentameric protein such as coproheme decarboxylase. The incomplete cleavage may make partially labelled particles escape binding to the affinity column and flow through together with oligomers having all His-tags cleaved. This is why for multimeric proteins, it is worth performing detailed analysis of the applicable ranges of eluting agent concentrations.

In the case of homo-polymeric particles, multiple enzymatic reactions must happen before His-tags are removed from all monomers (Figure 7). An incomplete cleavage of the His-tag for multimeric proteins may be difficult to mitigate by size exclusion chromatography. The second round of IMAC should retain both TEV protease and particles partially labelled with His-tag. However, IMAC purification conditions are frequently optimized for fully labelled proteins, e.g. binding buffers contain some level of imidazole to minimize non-specific binding, and even such low levels of chelators may be sufficient for escape of partially labelled multimers in flow-through fractions during the second round of IMAC. The size exclusion step that follows the second IMAC usually cannot separate species that differ only by the mass of the His-tag and its linker for macromolecular assemblies having larger masses than 100 kDa. This is an additional factor that should be considered when one analyzes His-tag interactions with the AWI.

If retaining the His-tag allows for more general modulations of preferred orientation, then this suggests additional biochemical strategies for modulating interactions with the AWI and modulating the preferred orientation that is driven by these interactions: for example, modifying the length and the nature of the linker between the His-tag or other affinity tags, mixing tagged and untagged proteins, attaching other decoy molecules, e.g. pegylation, or using reductive methylation of lysine residues (Means and Feeney, 1990; Rypniewski et al., 1993), to name just a few. Reductive methylation was used to change the pattern of hydrophobicity on the surface of proteins to promote their crystallization (Kim et al., 2008; Tan et al., 2014). One can expect that reductive methylation will have a similar impact on interactions with supports used in cryoEM SPR. Finally, one can also use a simple additive (e.g. 0.2 mM Ni_2_SO_4_) to adjust the state of His-tags in a labelled protein. This strategy was already successfully used in the case of membrane proteins, to change their associations with micelles (Rasmussen et al., 2019) and consequently change their aggregative properties. The Supplementary Figure S3 shows also another protein, for which we observed a dramatic change of the preferred orientation between sample with and without a His-tag.

Using tags as anchors and decoys to modify interactions with the AWI and consequently modulate patterns of preferred orientation offers an additional strategy to improve sample handling for cryoEM.

## Data Availability

The original contributions presented in the study are included in the text of the article and in the Supplementary Materials. The datasets analyzed in this study can be found in EMPIAR (deposits EMPIAR-10363, EMD-21373 and EMPIAR-10362, EMD-21376). https://www.ebi.ac.uk/empiar/EMPIAR-10363/, https://www.ebi.ac.uk/empiar/EMPIAR-10362/,https://www.emdataresource.org/EMD-21373/, https://www.emdataresource.org/EMD-21376. Further inquiries about data can be directed to the corresponding authors.
