# Supplementary material for: The His-tag as a decoy modulating preferred orientation in cryoEM
Source: Front Mol Biosci. 2022 Oct 17;9:912072. doi: 10.3389/fmolb.2022.912072 (PMC9619061; doi:10.3389/fmolb.2022.912072)
Supplement: Supplementary file 3 [file DataSheet1.docx]

**Supplementary Materials**

Fig S1. SDS PAGE:

SDS-PAGE for HemQ with His-tag entirely cleaved and HemQ with His-tag entirely present (Supplementary Figure S1A) was performed with Mini-Protean TGX 4-20% Stain-Free gel in the Tris-Glycine buffer system with the experiment run for ~30 minutes at 200 V constant current. The gel was stained with AquaStain (Buldog Bio). SDS-PAGE for HemQ with the His-tag partially cleaved (Supplementary Figure S1B) was performed with NuPAGE 4-12% Bis-Tris gel run with MES SDS running buffer for ~35 minutes at 200 V constant current. The gel was stained with AquaStain as well.

Fig S2. Mass Spectrometry:

Protein samples were analyzed by LC/MS, using a Sciex X500B QTOF mass spectrometer coupled to an Agilent 1290 Infinity II HPLC. Samples were injected onto a POROS R1 reverse-phase column (2.1 × 30 mm, 20 µm particle size, 4000 Å pore size) and desalted. The mobile phase flow rate was 300 µL/min and the gradient was as follows: 0-4 min: 0% B, 4-22 min: 0-55% B, 22-22.1 min: 55-80% B, 22.1-26 min: 80% B. The column was then re-equilibrated at initial conditions prior to the subsequent injection. Buffer A contained 0.1% formic acid in water and buffer B contained 0.1% formic acid in acetonitrile.

The mass spectrometer was controlled by Sciex OS v.1.6.1 using the following settings: Ion source gas 1 15 psi, ion source gas 2 25 psi, curtain gas 35, CAD gas 7, temperature 200 °C, spray voltage 5200 V, declustering potential 80 V, collision energy 15 V. Data was acquired from 1400-3600 Da with a 1 s accumulation time and 80 time bins summed. The acquired mass spectra for the proteins of interest were deconvoluted using BioPharmaView v. 3.0.1 software (Sciex) in order to obtain the molecular weights. The peak threshold was set to ≥ 5%, reconstruction processing was set to 20 iterations with a signal-to-noise threshold of ≥ 20 and a resolution of 2500.

The results for both samples are presented on the Supplementary Figure S1A (HemQ without His-tag) and on the Supplementary Figure S1B (HemQ with His-tag).

Fig S3. Electron micrographs:

Coproheme decarboxylase from *Geobacillus stearothermophilus* (HemQ) with and without His-tag is presented on the Supplementary Figures S3A (without His-tag), S3B (with His-tag partially cleaved), and S3C (with His-tag present in all copies. The conditions of proteins and grids preparations are presented in the text.

Glucose isomerase (GI) from *Streptomyces rubiginosus* with and without His-tag is presented on the Supplementary Figures S3D (without His-tag) and S3E (with His-tag). Both experiments were performed as described before (Bromberg, Guo et al. 2020).

Briefly, GI without His-tag was purchased from Hampton Research. Protein slurry was dialyzed three times against excess of dH_2_O, concentrated to ~40 mg/ml, applied to Quantifoil grid and imaged. GI with His-tag was expressed in *Escherichia coli* with the coding sequence of GI obtained as inserted into pET151-D-TOPO vector containing N-terminal 6×His-tag followed by the amino acid linker, the TEV cleavage site and a short sequence extending to N-terminal methionine residue of GI (**MHHHHHHGKPIPNPLLGLDSTENLYFQGIDPFT)**.

Bromberg, R., Y. Guo, D. Borek and Z. Otwinowski (2020). "High-resolution cryo-EM reconstructions in the presence of substantial aberrations." IUCrJ **7**(Pt 3): 445-452.
